# Linking repeated subjective judgments and ConvNets for multimodal assessment of the immediate living environment

**DOI:** 10.1016/j.mex.2024.102556

**Published:** 2024-01-05

**Authors:** Miroslav Despotovic, David Koch, Simon Thaler, Eric Stumpe, Wolfgang Brunauer, Matthias Zeppelzauer

**Affiliations:** a University of Applied Sciences Kufstein Tirol, Kufstein, Tyrol, Austria; b University of Applied Sciences Sankt Poelten, Lower Austria, Austria; c Data Science Service GmbH Vienna, Vienna, Austria

**Keywords:** Location analysis, Elo rating, Hedonic pricing, Computer vision, Affective content analysis, Alternative forced choice, Assessment of the quality of the immediate living environment

## Abstract

The integration of alternative data extraction approaches for multimodal data, can significantly reduce modeling difficulties for the automatic location assessment. We develop a method for assessing the quality of the immediate living environment by incorporating human judgments as ground truth into a neural network for generating new synthetic data and testing the effects in surrogate hedonic models. We expect that the quality of the data will be less biased if the annotation is performed by multiple independent persons applying repeated trials which should reduce the overall error variance and lead to more robust results. Experimental results show that linking repeated subjective judgements and Deep Learning can reliably determine the quality scores and thus expand the range of information for the quality assessment. The presented method is not computationally intensive, can be performed repetitively and can also be easily adapted to machine learning approaches in a broader sense or be transferred to other use cases. Following aspects are essential for the implementation of the method:

• Sufficient amount of representative data for human assessment.
• Repeated assessment trials by individuals.
• Confident derivation of the effect of human judgments on property price as an approbation for further generation of synthetic data.

## Graphical abstract

**Figure:**
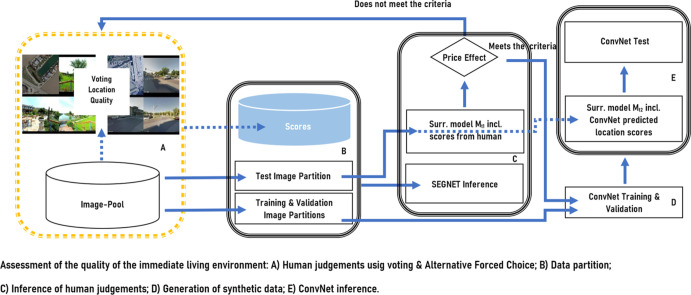

Specifications table

**Table:** 

Subject area:	Economics and Finance
More specific subject area:	Regional economics
Name of your method:	Assessment of the quality of the immediate living environment
Name and reference of original method:	NA
Resource availability:	NA

**Method details**

## Introduction

The environment of residential buildings has a considerable impact on the well-being of the occupants and therefore also influence real estate prices. The authors in [1] have summarized 470 possible determinants for hedonic price estimation of real estate that have been used in the scientific literature so far. Among them, 83 represent location and nature features. Within mentioned categories, there are variables that represent features of perceived attractiveness of a neighborhood, such as the view [[2], [3], [4]], the appearance of the environment [5,6], or abstractly derived attributes, such as the image of the neighborhood [7]. Other variables that affect neighborhood quality include measurable features such as plot shape and size, nearby amenities, and other proximities [8] among others. For a literature review on the effects of the view of a property on its price see [[1], [9]]. Especially the green area around real estate has a positive effect on prices [10] and visual interaction or contact with green space increases the location attractiveness [11]. This includes the level of tree cover in the immediate environment as well as in the visible environment, the lawn coverage in the vicinity and elements such as the presence of hedges, flower arrangements or rock plants [12]. As the visual appearance of neighborhoods and buildings can be captured in images and access to such imagery, especially in the form of Street View images [13] and real estate listings has become more readily available, these can be used in the automated assessment models, thus potentially improving the overall prediction accuracy [14].

**Fig. 1:**
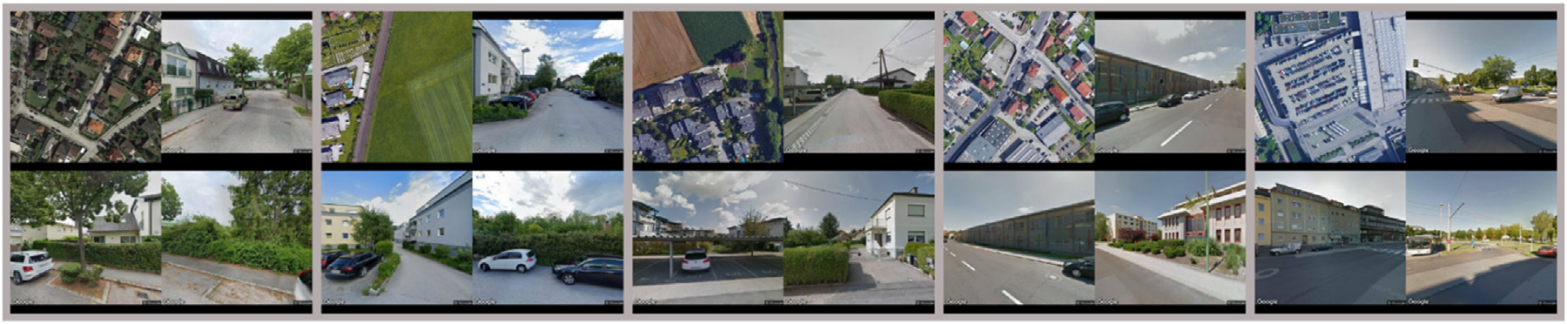
Selection of exemplary image montages used for the experiment representing the immediate environment of the apartments [13].

Both conventional valuation and marketing of real estate are highly dependent on the objective assessment of its qualitative characteristics. This assessment is a demanding and often time-consuming task which requires individual expertise. Furthermore, due to the individual assessment by mostly only one trained expert, the necessary criteria like the quality of location, condition or interior design of the building are influenced by subjective distortions such as e.g. personal preferences, suggestiveness or individual perception. With automated methods of Computer Vision, it should be possible to arrive at less subjective assessments of real estate features if the data used to train the supervised Computer Vision algorithm (ground truth) is less biased, assuming that the annotations to the image content are less subjective. Therefore, the images have to be annotated manually, which for an individual is usually not a problem when it comes to objectively measurable single features such as the presence of a motorway in the vicinity or the identification of a nearby park. But as soon as it comes to human interpretation of multiple visual semantics the annotation of a single person may introduce a subjectivity bias in the overall dataset (see example images in Fig. 1).

We consequently hypothesize that the predictions of location quality are less biased if the annotation is made by multiple independent persons. Additionally, repeated estimation trials of one individual should also lower the total error variance and, following that, the range of expectation of the estimates should be narrowed yielding more robust results. Overall, this presents us with the difficult task of finding a method that allows for repeated independent evaluations and provides estimations that are reliable enough to use for assessing site quality. Hence, the main contribution of this work is the development a research design for the assessment of location quality that is not computationally demanding, obtains reliable estimates and facilitates independent repeated visual assessments by various persons. Our approach is essentially based on the idea from [15]. However, in the present method, the experimental setup is methodically consolidated, applied in more depth and to a different use case and inferentialised by the use of additional evaluation tool.

To get the most objective annotations of attractiveness for images of the environment of a residential property the basis of our assessment tool is the Elo rating system [16], which is well known for rating chess players. Since a human can hardly judge the attractiveness of a single image without one or more reference examples, we rely on pairwise comparisons of images using Alternative Forced Choice [17] and backend calculation of quality scores. After the annotation by human subjects, we train a Computer Vision algorithm, more precisely a Convolutional Neural Network (ConvNet), on the dataset of images and the cumulative assessment score as ground truth. The ground truths and the ConvNet predictions are then tested for their usability in a hedonic pricing models to examine their effect on the real estate price. Based on this, it should be possible to predict the attractiveness of the nearby environment from input images without the need for expert or other single judgment, since we do not need manual annotation once we have ground truth for the visual data.

## Literature review

### Quality assessment of the real estate location

According to [18] the link between consumers to urban areas can be measured on three levels concerning spatial range: the building or house, the neighborhood and the whole urban area. Their work finds that people in cities are more attached to their home and the city itself than to the neighborhood they live in. Their research generally puts more focus on immediate neighborhood variables. The immediate environment of a property and its impact on the property price has already been studied in the 1980s where the view or visual quality, the proximity to streets, the impact of polluting industries or commercial districts were investigated [19]. Later, [20] found that environment variables derived from small scale 30 m statistical grid raster can be used in hedonic models. [21] shows in a data-driven study with street and satellite images using a Computer Vision algorithm, that these types of images are able to capture such urban qualities and improve models for estimating house prices.

An important determinant in the building environment that can be obtained from images is vegetation such as the count of trees. There, research finds an influence on sales prices [22,23]. Additionally, street greenery attracts higher income people, thus, serving as a signal for property attractiveness and higher life quality [19]. It also has a positive impact on environmental quality, which in addition makes people willing to pay more for sea-view, road connection, the proximity to a park [24], to water bodies and recreation areas [25]. Parks and tree cover in general add utility to the neighborhood area [26]. The visual appearance of the environment, which can also be derived from photographs, and its perception by the residents are closely linked. For instance, the perceived safety of a neighborhood has been predicted in [27] by using Google Street View images from the immediate environment of a property and the online survey data. Furthermore, the perceived safety of a neighborhood impacts human activity in the neighborhood [7]. In their study, again greenery and windows that face the street are the main contributors to a safe perception. This is in line with the broken window theory which states that visible signs that are evidence for social disorder or crime further increase the likelihood for more social disorder or crime [28]. In this regard also [29] finds that disorder in a neighborhood negatively impacts housing sales prices. A remote sensing approach is conducted by [30] where they find that there is a correlation between availability of green areas, swimming pools, pavement conditions and/or sports facilities extracted from satellite data and the socioeconomic profile in a small-scale neighborhood of the city of Lima. The paper of [31] uses a similar methodology and finds that it is possible to predict the settlement of university graduates, as a proxy for location or neighborhood quality, on a 250 m x 250 m small scale grid of satellite pictures. Also, the density of housing can be assessed in the view from a property: here [32,33] find that with an increased density, housing prices decline. Therefore, we also hypothesize that the view from a residential building into the neighborhood has an impact on real estate prices.

### Media content analysis

The measurement of visual stimuli in many research areas is often based on human emotions and subjective decision making as in [[34], [35], [36]]. The capability of humans to perceive high affective and cognitive semantics compares to the computer's ability which is only recognizing visual content of the lower level. The idea of the assessment of pictures in this paper is based on the works of [37,38]. The literature shows that an observer comes to different comparative judgements when confronted with the same pair of stimuli. Therefore, it can be assumed that a subject that has to decide on the attractiveness of two visual stimuli will be inconsistent when confronted with the same visual comparative judgement on an additional occasion. Borrowing the ideas outlined in [39], we also assume that certain emotions of humans will exert impact on the evaluative judgement. The field that focuses on the analysis of media content stimuli such as videos, images and audios and their impact on reactions and other emotional expressions of the receiver is called Affective Content Analysis (ACA). The clear focus in the ACA literature is on discrete and dimensional maps of emotions. In this papers' case, the emotions represent the positive or negative arousal to visual stimuli from the view on the immediate environment which is usually expressed by like/dislike, approve/disapprove, etc. [38].

In broadcasting research, affective media content experiments have been used for decades [40]. The experimentation started in the 1970s, where broadcasting companies developed the first concepts which incorporated an automated assessment process. In that process, the inference on the effect of affective media content was measured by collecting test results from subjective human based assessment experiments but also objective computer-based trials [41]. The many areas where ACA has been applied up until today range from Affective Computing [[38], [42]] over Sentiment Modeling [43] to Human-Computer Interaction and Image retrieval [44]. A methodology that is complementary to ACA is Alternative Forced Choice (AFC) [17]. It is more effective in inferring with human stimuli which stem from visual media content interaction. In essence it is based on a concept called multi-alternative perceptual decision [45]. Therefore, when a subject is forced to choose between multiple alternatives, different scenes or objects in that scene can be tested on their influence, even when all elements differ visually but still sharing a common category and properties. Alternative Forced Choice experiments, in the context of Affective Content Analysis, have been used for recognizing emotions in faces [46] or to assess color preferences of individuals [47].

### Hedonic pricing in automated valuation

Computers have been used in real estate valuation since the 1970s [48] where its initial name was automated valuation system [49]. Nowadays, automated valuation systems (AVS) are defined as data analysis tools with a single or multiple AVMs together with a user interface. The AVS tool can then be used to assess the price of an individual property or a parcel of land which is based on a mechanism for structured decision making [50]. In such a tool box, the AVM predictions are reliant on the input data, the analysis approach and the employed method.

The mostly used method for automated valuation models is the hedonic pricing method (HPM). It is a very versatile and flexible method which can easily be applied to a variety of different data. Furthermore, it can be combined with different other automated valuation methodologies such as market or income approaches, probabilistic and non-probabilistic approaches. The hedonic modeling approach states that a good consists of many different characteristics which all can affect its total value or price [8,24]. Therefore, hedonic modeling analyses the different characteristics and its marginal effects on the total price where the marginal effect represents the change in the price associated with a one-unit increase in the predictor variable. For a literature review on the common factors influencing the price of real estate see [1] resp. for the literature summary regarding hedonic pricing see [[6], [51]].

### Deep learning for image pattern recognition

Most artificial intelligence applications like convolutional neural networks (ConvNets) are based on Deep Learning as their theoretical foundation. Here the basics for their functionality is on approximation and optimization but also on the paradigm of representation learning (for a review see [52]). This is due to the fact that most predictions are founded on the training of the algorithm on a previously annotated dataset, the ground truth. More specifically, ConvNets optimize decision weights in hierarchical layers where the representative patterns of the input image are recognized. Besides training a ConvNet from scratch, a very common training technique for visual intrinsic feature classification and regression is the so-called transfer learning. Here, a network already pre-trained on specific data is trained on the data from the same domain, resp. some layers of the network can be unfrozen and thus the parameters can be fine-tuned by new input signals [53].

To come to a holistic price estimate of real estate a multitude of different modalities of data have to be considered. Specifically, in the sphere of real estate besides tabular data, visual data definitely contributes to the price development. To automatically include visual data into the pricing formula, the use of Computer Vision Machine Learning algorithms is currently the most promising methodology [31]. By using ConvNets the visual content in real estate (e.g. front view pictures or pictures of the interior) can be translated into tabular data, but, as convolutional neural networks are considered black-box algorithms, it is important to understand the explainability of those models and the extracted intrinsic and complex visual features. A common method to explain the performance of black box models and to make the predictions explicit is the application of interpretable surrogate models [54,55]. This kind of model tries to find explanations for the predictions of a black box model by either in a direct way i.e. comparing the test results (predictions) of two models with the same test data or in an indirect way where the extracted information that stems from the black-box model is included into a model that is meant to be used and then investigate the significance of the gained information [[56], [57], [58]]. Until now, ConvNets were mainly used for classification [7,[59], [60], [61]]. A real estate related example is the article of [62] where the authors classify human emotions that arise when subjects see property pictures to include the detected emotions in a valuation model, or the articles of [63] or [64] where a ConvNet is evaluating the impact of the exterior resp. the interior look of a building on prices. Regarding the location evaluation [21] use street view and satellite images in a ConvNet model to predict the visual desirability of a neighborhood. They generate a scalar output from predictions for the estimation of real estate prices in a hedonic model. They observed an effect of the attractiveness of a neighborhood on the price.

The use of ConvNets for regression is not as widespread as for classification problems. One of the early works incorporating ConvNet for a regression task is on predicting angles [65], distances for 3D pose estimation [66], age estimation [53], or the grasp pose of objects [43]. Another paper is using a regression ConvNet to predict the coordinates of the position of a robot in a building [67]. Regression ConvNets also are important in autonomous driving, e.g. in [68] for the prediction of the vehicle's current position. The methodology is also used for predicting stock prices via annual reports and text analysis [69] and using historical data [70]. In the real estate field, which as stated above, is a showcase field for coupling Computer Vision and valuation, [71] use a spatial regression ConvNet for real estate valuation. [72] choose a pretrained Xception ConvNet to predict the apartment rent prices using pictures of the floor plan as the input. [73] also use a ConvNet to augment real estate appraisal using images. They use pictures of the exterior and interior, but conclude that pictures alone are not sufficient to produce a reliable price estimate. Thus, they fused the images with additional textual data of the property which constituted the new input for the ConvNet and provided more accurate results. Using a GoogLeNet ConvNet and the combined modalities, the two baseline models solely using text or images were outperformed. [62] also use a regression ConvNet to predict rental prices in Wuhan neighborhoods based on the spatial density of points of interest. The authors find that a regression ConvNet outperforms other prediction methodologies. Furthermore, [74] use a ConvNet with photographs and other metadata of houses improving the predictions of house prices, while stating that their methodology incorporates the workmanship into the valuation process. A similar study which is based on a ConvNet but using only textual data yields better results compared to traditional house pricing methods [75].

## Overview of proposed method

**Fig. 2:**
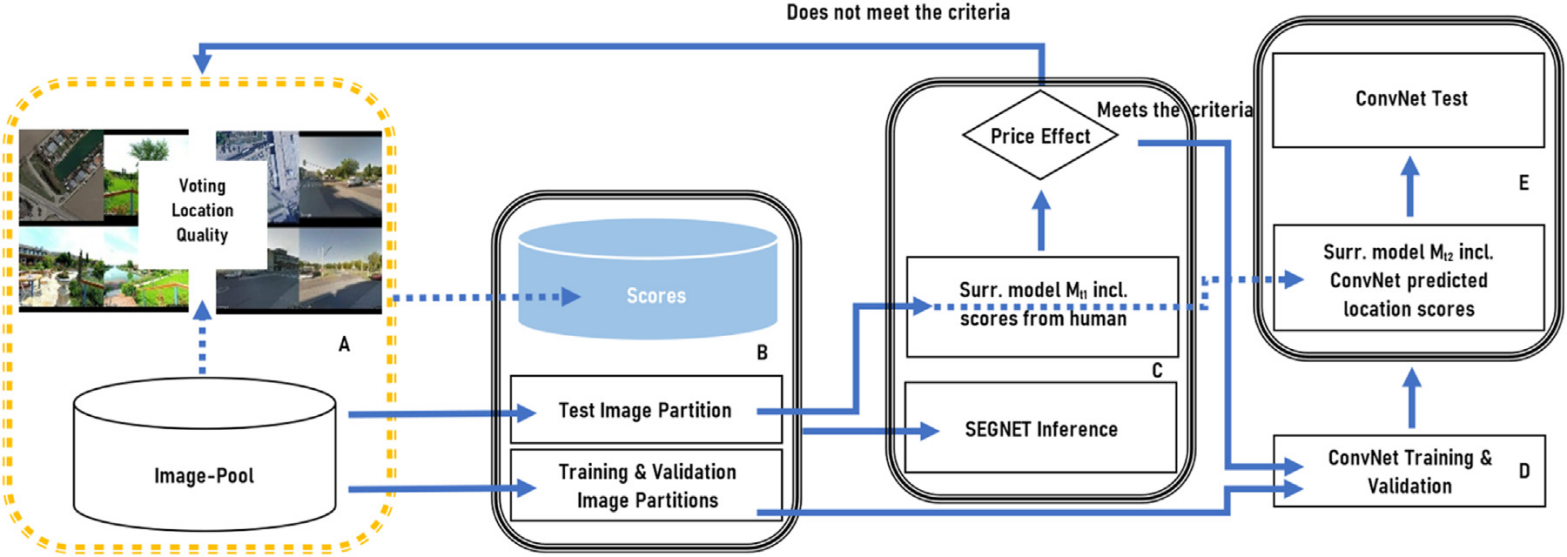
Methodical steps illustrated in the proposed order. (A: Human judgements using voting & AFC; B: Data partition; C: Inference of human judgements; D: Generation of synthetic data; E: ConvNet inference.

Our approach focuses on the subjective estimation of location quality, with the obtained estimates being incorporated into a hedonic model to assess their impact on the property price. In a separate experimental stage, the estimates are learned from a neural network to automatically replicate resp. synthetically generate human estimates and use them in an automated valuation model. To better clarify the applied approach, we have summarized its theoretical basis in the following Section. Fig. 2 shows the methodological steps in proposed order.

### Elo rating (method steps A & B)

In a qualitative assessment based on the classification, the main problem lies in the unavoidable fuzziness of the decision boundaries between individual classes. Consequently, the implementation of subjectively estimated quality classes in the price prediction of a real estate can have a significant impact on the estimated sales or rental price. This is problematic, because it implies that detailed description lists and a lot of experience on the part of the judging person are required to adequately estimate the quality of a real estate characteristic. To overcome this problem, we apply a system in which probands are shown successively pairs of randomly selected image montages from a pool with N representative images and have to decide which of the two montages has the higher quality of the immediate environment of the apartment building (each montage references to the immediate environment of an apartment; see example image montages in the Fig. 1 in Section 2).

By using such subjective serial pairwise comparisons of image features, we can estimate a perceptual counterpart of the features as a scaled collection of stimuli (expressed by a metric score and not a class!) that includes simultaneous as well as successive contrasts due to application of repeated trials. To this end, we perform Alternative Forced Choice [17] and thus force probands to choose between three alternatives whereby the responses are recorded by an automatic backend scoring system. The proposed approach is expandable and requires sufficient number of probands, representative images as well as repeating of trials in order to decrease the error variance continuously and thus gain usable data. To measure the quality of two directly compared images in a continuous manner, assuming that their estimated values will vary adequately in the data distribution, we apply the Elo formula. The Elo rating system is used in practice to quantify the relative abilities of chess players [16]. At Elo rating, each player starts with an initial score and depending on how he/she plays against players with higher or lower Elo his/her score is being updated according to Eqs. (1) and (3). Definition for expected Elo value of a win for the problem at hand: If image A has rating R_A_ and image B has rating R_B_, then the expected value of image A beating image B is given by [76]:

(1) $$Pr\left(A\;>\;B\right)=E_{AB}=\frac{1}{1+10^{\frac{R_{A}-R_{B}}{400}}}\;$$

where the initial value for R is set to 1500. Hence, note the logistic property:

(2) $$E_{AB}+E_{BA}=1$$

The parameter 400 in the 2nd equation controls the different probabilities of the possible outcome for the higher and lower rated image. Having an expected score for image A when voting against image B and three possible outcomes for A, namely win, lose, or draw, which correspond to values of 1, 0, and 0.5, we calculate Elo score as follows:

(3) $$R_{A}^{\prime }=\left\{ \begin{array}{c} R_{A}+K_{A}\left(1-E_{AB}\right),ifA\;wins\\ R_{A}+K_{A}\left(\frac{1}{2}-E_{AB}\right),ifA\;draws\\ R_{A}+K_{A}\left(0-E_{AB}\right),ifA\;loses \end{array}\right.$$

The constant K (*K* = 10) is used to adjust the weighting sensitivity of the scores update. Based on [77], the Elo algorithm is assumed to be sufficiently robust to represent the rating results with reasonable proportionality

### Hedonic regression (method steps C & E)

To evaluate the effect of estimated Elo scores on the real estate price, we use Hedonic Price Method. HPM is also known as Hedonic Regression and is commonly used method to predict real estate prices by estimating the marginal contribution of real estate characteristics to the price. In general, the hedonic price function has the form

(4) $$P_{i}=f\left(Z_{i}\right)$$

where P is the price of the real estate and f is a function of the vectorized characteristics Z of the real estate. The basic assumption in HPM is that the relevant determinants of the dependent variable (price or index) are known in advance. In practice, different variables are used depending on the research question, the preference of the researchers or the availability of data [51]. If we divide the property characteristics Z into three main subcategories then the price function has the form:

(5) $$P_{i}=f\left(S_{i};L_{i};N_{i};E_{i}\right)$$

where S_i_ is a vector of structural real estate characteristics, L_i_ is a vector of location variables, N_i_ represents neighborhood characteristics and E_i_ represents characteristics of immediate building environment [1]. A prior perceptible effect of the estimated location scores on the price is also the main criterion for generating synthetic data (See next subchapter).

### ConvNet regression (method steps D & E)

**Fig. 3:**
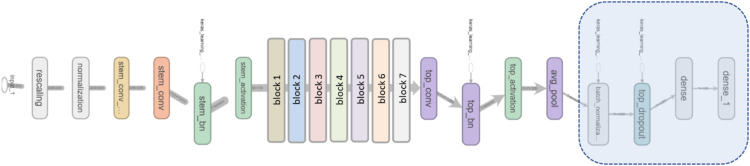
Simplified representation of EfficentNet-B0 architecture with modified top layers for regression task (layers on the far right).

It is essential to verify whether human estimated scores can be learned from a ConvNet and be generalized to new images unknown to the trained network. For the replication, we use the EfficientNet neural network [78] for the regression task. EfficientNet is able to uniformly scale width, depth, and resolution of the network allowing for adaptive scaling and thus for models with different input sizes and floating-point operations (FLOPS). The structure of the base model EfficentNet-B0 (see Fig. 3) consists of seven building blocks with inverted residual blocks - also called Mobile Inverted Bottleneck (MBConv) and stem layers. Stem layers have the function of a compression mechanism that leads to a fast reduction in the spatial size of activations, and therefore reduce storage and computational costs. MBConv blocks use inverted residual blocks with depthwise separable convolution, which on the other side significantly reduces the number of parameters. Each MBConv includes a Squeeze and Excitation (SE) sub-block which dynamically assign high weights for more important channels and so map channel dependence while also providing access to global spatial information of the input signal. With the EfficentNet, it is possible to control and optimize the trade-off between accuracy and FLOPS.

## Setup for the assessment of the immediate living environment

In line with the proposed workflow (see Fig. 2) the following section outlines detailed description of the data and individual experimental steps. For better understanding, we refer to the human estimated values for the quality of the immediate environment in the following subchapters predominantly as location scores.

### Objectives of the experiments

Our objective is to test the extent to which the quality of the immediate environment of a property can be assessed on the basis of photographs through repeated comparative assessments by individuals. Having identified an effect of human-estimated quality scores on the price of real estate as the main criterion for the synthetic data generation, we are testing to what extent a ConvNet regressor is able to learn relevant location information from the associated images and to transfer that knowledge to new unknown data. Furthermore, we perform additional tests: (a) Comparison and analysis of human-estimated scores and Convnet-predicted scores to identify (non)linear relationships to micro-location in order to better infer the results , (b) Verification whether more training data improves the performance of the ConvNet, (c) Inference to what extent different visual contents (sky, buildings, streets, pavements, green areas, fences, cars) in the images correlate with the human estimated location scores. These tests are hereafter referred to as additional tests.

### Initial data

For the subjective estimation we use the initial set *Ɗ_i_* with 1044 representative image montages each including three street view images showing the environment left, right and vis-à-vis of a unique building with the apartment of interest. The building resp. the building's position is unknown for participants. The fourth image in the montage represents orthogonal view of closer environment of the building. The actual position of the building within the orthogonal view is not always in the center of the image, but varies so that the participants do not get any clues about the actual building. The images are taken from the real estate advertisements with properties in several Austrian provincial capitals, including Vienna, as well as few smaller Austrian cities, so that a certain diversity in the appearance of the architecture and landscape can be achieved. All image montages from *Ɗ_i_* correspond to 1044 instances (apartments for sale) in dataset *P_i_* which includes real estate structural and location characteristics *S_i_, L_i_, N_i_*.

### Setup for human judgments

**Fig. 4:**
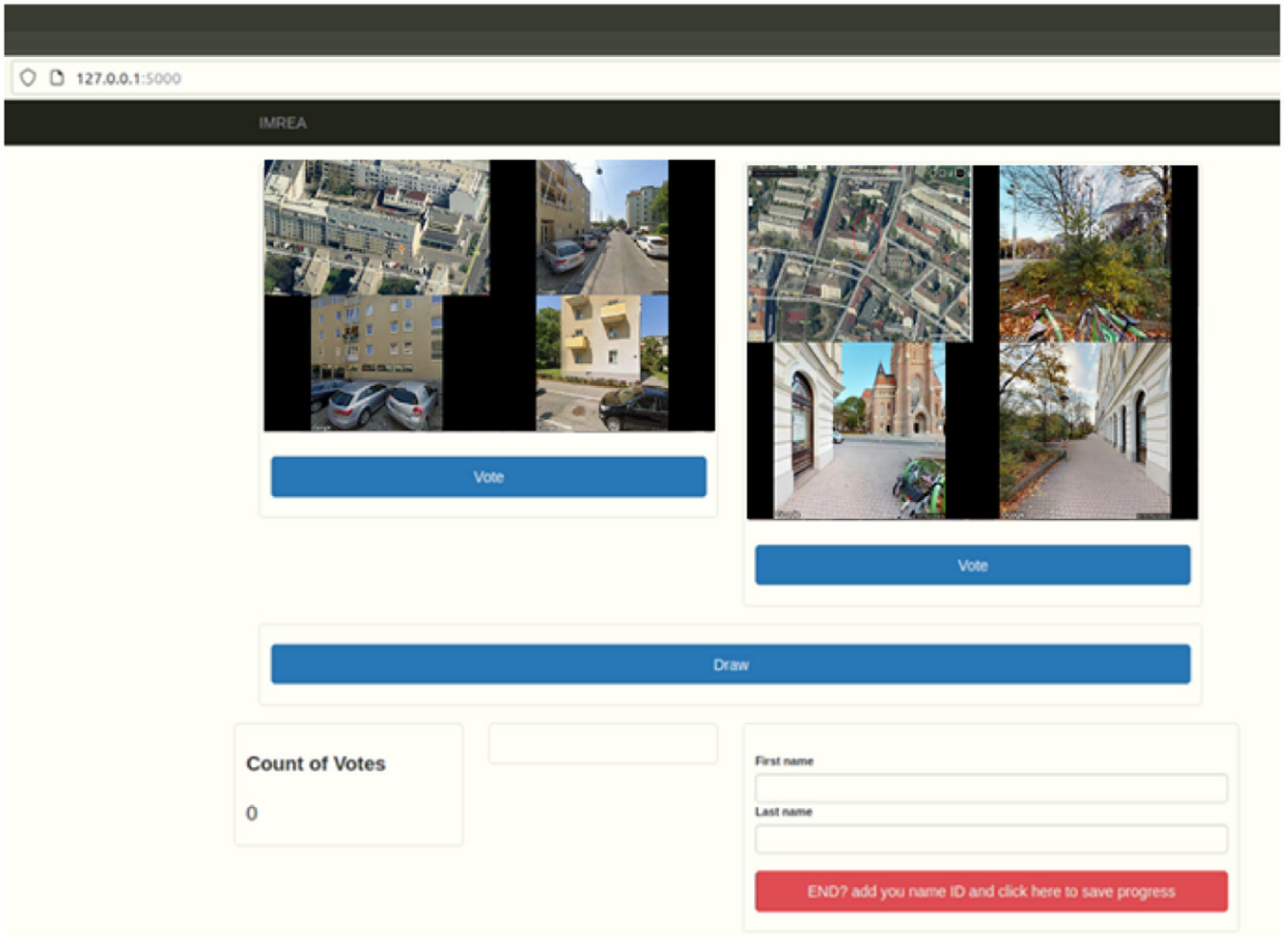
User interface of the voting app for subjective judgments.

For the human subjective judgments of the immediate environment of apartments we use modified browser-based application [79] based on Python modules Flask as underlaying framework and Sqlite for data storage. By using the application's GUI (see Fig. 4), the participant is able to vote between two displayed image montages which of the two represents better quality of the immediate environment around a building. For every vote and for each of montages a quality score is calculated backend based on Elo rating (see Eqs. (1) and (3) resp. in accordance to resulted threefold forced choice: win for one of the both montages or draw for the displayed montage pair.

For each voting round with a single participant, 522 pairs are randomly selected from the initial pool *Ɗ_i_* with 1044 representative image montages. The overall voting took 36 rounds with 6 participants and 6 repeated trials per person whereas each person could repeat the trial only after the remaining 5 persons have voted once. In principle, the voting process has no limit respectively it is assumed that the total error of the estimates decreases the longer the voting lasts and the more participants are involved. After each voting round all estimated Elo scores are saved into a separate database table resulting finally in a final data set *S_i_* with 1044 scores for each image montage. Note that not all combinations of image pairs can be carried out, since this would result in 544,446 possible combinations (acc. *N*(*N*-1)/2) out of 1044 image montages used.

### Effect of human estimated location quality scores on the real estate price

At this stage, we integrate location scores into an applicable valuation model and investigate the significance of the gained information as the examination of the admissibility criterion for the generation of synthetic data. We first union *S_i_*, and *P_i_* and then determine the ratio for the training, validation and test sets (75:12:13). Then we subset a test data *T_h_* using a predetermined random seed *rs* resulting in a data set with 138 test instances. We use test set *T_h_* to estimate target variable apartment price in a hedonic model M_t1_ including estimated location scores along with the structural apartment characteristics living space, fictive year of construction, balcony, loggia, garage, and location characteristics micro location quality, and macro location (city) as predictor variables. Thereby, the explanatory power of the models and the effects of all location predictors are inferred.

Hereby, we control model for the micro and macro location quality, which permits a more conclusive inference on the effect of estimated location scores. We also take emphasis on the age and the condition of the building itself because the effects of an investment or renovation of a property have large impact on the building's remaining useful life and these effects can be represented more realistically in a price model with the fictive year of construction [80].

Tables 1 and 3 show four different settings of the base model M_t1_ and of the model with predicted location scores *M_t2_*. In the 1st setting (base setting) the response variable was regressed without any location variable. In the 2nd setting, location scores are added, in the 3rd setting the model is controlled for the variable micro location quality (estimated by experts), and in the 4th setting we add macro location represented by the city. Reference levels for variable city and micro location (not shown in regression outputs in Tables 1 and 3) are city of Graz and micro location with simple quality. In all settings the variable living area and the response variable price/m² are log-transformed.

**Table 1:** Performance of the M_t1_ model with log-transformed response variable (price/m²) and four settings: without (1) and with (2) estimated location scores, controlled for micro (3), and macro location (4) including the coefficients, significance asterisks and standard errors of the variables.

	Setting 1	Setting 2	Setting 3	Setting 4
Intercept	9.542 (***)	9.545 (***)	9.241 (***)	9.017 (***)
	0.257	0.254	0.237	0.188
log(living area)	-0.481 (***)	-0.483 (***)	-0.452 (***)	-0.420 (***)
	0.057	0.056	0.051	0.04
poly(fictive yoc, 2)1	0.599	0.549	0.321	0.640 (*)
	0.364	0.361	0.326	0.269
poly(fictive yoc, 2)2	0.765 (*)	0.842 (*)	0.379	0.492
	0.351	0.348	0.322	0.255
garage	0.175 (*)	0.185 (**)	0.184 (**)	0.148 (**)
	0.069	0.068	0.061	0.049
balcony	0.179 (**)	0.181 (**)	0.144 (*)	0.083
	0.062	0.061	0.056	0.045
loggia	0.107	0.113	0.09	0.086
	0.072	0.071	0.064	0.051
poly(location scores, 2)2		0.717 (*)	0.939 (**)	0.633 (**)
		0.317	0.287	0.231
good micro location			0.247 (***)	0.272 (***)
			0.058	0.046
very good micro location			0.368 (***)	0.444 (***)
			0.067	0.054
city Innsbruck				0.396 (***)
				0.07
city Klagenfurt				0.170 (**)
				0.06
city Linz				0.137 (*)
				0.059
city Salzburg				0.363 (***)
				0.062
Adj. R^2^	0.364	0.385	0.503	0.693
Adj. R^2^ w/o location scores		0.364	0.467	0.518
Num. obs.	138	138	138	138

Since we do not expect a large price premium for the scores, we focus our analysis of the results primarily on the significance of the estimated location scores under the expectedly large effect of the micro and macro location. In this context, it is important to observe to what extent the effect of the location scores is resistant to the other location variables, resp. whether the scores are in a significant range and if so, continue to remain even if we add micro and macro location variable. In the base model M_t1_ in the Table 1, the parameters of the variable location scores in the 2nd are significant indicating an effect on the response variable and improving the model for 2.1%. According to D'Agostino skewness test for this variable, there is no necessity for a log transformation, however, the variable (living area) shows much stronger skewness (*p* = 2.2e-16) and was therefore log-transformed for all model settings.

Analysis with Loess locally weighted regression for location scores and apartments price indicates similar polynomial effects. Also, the variable fictive year of construction shows a non-linearity, and thus we apply orthogonal polynomials for location scores and fictive year of construction in the model. The location scores show small positive correlation with the response variable (0.14), but as mentioned, the relationship between the variables could be nonlinear and the location scores can affect the relationship with other parameter estimates, thus showing potential interactions in the model.

When we control the model for the variable micro-location quality in the third setting, we find conclusive effects as the significance of the location scores became higher despite the strong effect of the included micro-location. This is also evident from the difference in the goodness of fit of the model between the setting with both location variables (0.491) and the setting with only the micro-location (0.467). This confirms that there is additional information content in the images respectively estimated location scores what clearly indicates a good approximation. The strongest location effect occurs in the fourth setting with the macro location, at which location scores remain in the same significance range as in the previous setting and their standard error decreases. In fact, we note a clear successive improvement with each setting of the model, both in the significance of the scores and also of the model that enables the subsequent step of generating synthetic data.

### Training of convnet using human estimated location quality scores

We union *Ɗ_i_*, and *S_i_* and partition data by using same predetermined random seed *rs* in training *T_r_*, validation *V_l_* and test *T_s_* sets with the ratio of 788:118:138 instances. Thus, the scores from *T_s_* are the same location scores as in test set *T_h_*, resp. associated to same instances of *P_i_*. Using training set *T_r_* and validation set *V_l_* with image montages and estimated location scores as training data, we assign location scores as the response variable and associated image montages as predictor variable and train the EfficentNet-B0 [78] for the regression task. For training, we first crop the montages to a square shape, starting from the center of the montage maximizing the dimension by either the original height or width. Then the montages are scaled to 224 × 224 according to the input dimension of the EfficientNet0 network.

We adapted model for the regression task by adding batch normalization layer, dropout layer and regression output layer on the top and by modifying last dense layer to 1 neuron (see Fig. 3). As model metric for validation and training we apply mean-squared-error (MSE) loss and root-mean-squared-error (RMSE).

For the training we set training parameters as shown in Table 2. We limited the image augmentation to horizontal flipping only, as additional augmentation had a negative impact on the network performance. We let the ConvNet converge more slowly, thus reducing the learning rate on plateau when the loss stops decreasing, starting from 0.001 to a minimum learning rate of 0.0001. We do not apply early stopping for regularization but select the training stage with the best performance.

**Table 2:** Settings applied for the ConvNet training.

Optimizer:	Adam	Learning rate:	0.001
Batch size:	16	Epochs:	1000
Augmentation:	Horizontal flip	Reduce learning rate on plateau:	• min lr 0.0001 • factor 0.2 • patience 21
Top dropout rate:	0.3		

### Effect of convnet predicted location quality scores on the real estate price

In the next step, we aim at evaluating the location scores predicted by ConvNet in a hedonic model. For this purpose, we replace human estimated location scores in the test set *T_h_* with ConvNet predictions and setup hedonic model M_t2_ having exactly the same evaluation settings as for the model M_t1_ including predicted location scores along with the variables living space, fictive year of construction, balcony, loggia, garage, and location characteristics micro, and macro location (city). Thus, we infer for the model significance, for the significance and effects of the variables with an emphasis on the effect of the predicted location scores on the target variable apartment price.

To infer the generalizability of the ConvNet trained on the training set *T_r_*, we predict the location scores with the trained network on the test data set *T_s_* and include the predictions as a predictor variable in the M_t2_ hedonic model, applying exactly the same evaluation settings and having exactly the same data for the remaining features as for model *M_t1_*.

Table 3 shows the four settings of the surrogate model M_t2_, with included predicted location scores. By employing the predicted scores into the model, the model's goodness of fit increases slightly by 1.8%, showing an effect of the predicted scores. The 3rd setting shows similar effect of the micro location as in the model M_t1_ whereas the significance of the predicted scores in the model M_t2_ even increases. We note that the overall goodness of fit in the 3rd setting is smaller than in the 3rd setting of the model M_t1._ Including the macro location in the 4th setting improves model performance by nearly 19% compared to the previous setting, but to some extent eliminates the effect of predicted scores. Thus, the standard error of the scores decreases but the p-value increases slightly and remains however within the significant range. On the one hand, this shows that the network can detect additional information in the images and demonstrates a good approximation, but on the other hand, there is also evidence of a certain loss of information due to the inferior generalizability of the network, which is also reflected in the test RMSE value (39.547). This is largely due to the amount of training data available, as confirmed by the results of the additional test with the ConvNet training with different amounts of training data. However, in all 3 M_t2_ settings with the ConvNet predicted scores a clear effect can be observed.

(a) Additional Test: Comparison and Analysis of Human Estimated Scores and Convnet Predicted Scores

**Table 3:** Performance of the M_t2_ model with log-transformed response variable (price/m²) and four settings: without (1) and with (2) ConvNet predicted location scores, controlled for micro (3), and macro location (4) including the coefficients, significance asterisks and standard errors of the variables.

	Setting 1	Setting 2	Setting 3	Setting 4
Intercept	9.542 (***)	9.544 (***)	9.264 (***)	9.001 (***)
	0.257	0.256	0.24	0.191
log(living area)	−0.481 (***)	−0.484 (***)	−0.456 (***)	−0.418 (***)
	0.057	0.056	0.052	0.041
poly(fictive yoc, 2)1	0.599	0.706	0.475	0.819 (**)
	0.364	0.368	0.337	0.28
poly(fictive yoc, 2)2	0.765 (*)	0.786 (*)	0.326	0.45
	0.351	0.348	0.327	0.257
garage	0.175 (*)	0.174 (*)	0.170 (**)	0.138 (**)
	0.069	0.068	0.062	0.05
balcony	0.179 (**)	0.184 (**)	0.146 (*)	0.088
	0.062	0.061	0.057	0.045
loggia1	0.107	0.139	0.116	0.107 (*)
	0.072	0.073	0.066	0.052
poly(predicted scores, 2)2		0.771 (*)	0.819 (**)	0.534 (*)
		0.324	0.295	0.235
good micro location			0.241 (***)	0.268 (***)
			0.058	0.046
very good micro location			0.346 (***)	0.427 (***)
			0.067	0.054
city Innsbruck				0.391 (***)
				0.07
city Klagenfurt				0.157 (*)
				0.061
city Linz				0.143 (*)
				0.061
city Salzburg				0.396 (***)
				0.064
Adj. R^2^	0.364	0.382	0.491	0.688
Adj. R^2^ w/o predicted scores		0.364	0.467	0.518
Num. obs.	138	138	138	138

**Fig. 5:**
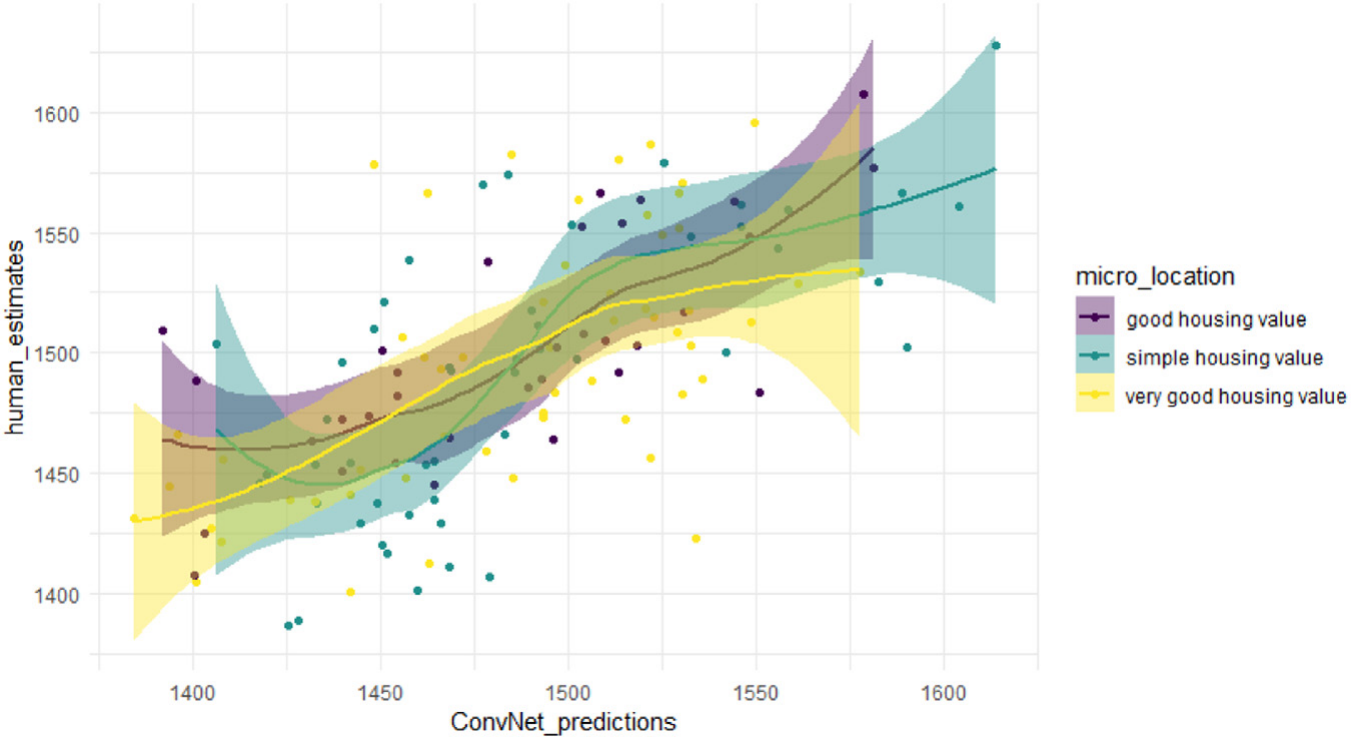
Scatter plot of estimated and predicted scores, grouped by the quality of micro location.

We examine in the first step selected statistics of interest for the location scores as well as for the location scores predicted by the ConvNet. Fig. 5 shows the scatter plot for the estimated and predicted scores. Starting from an initial Elo score (*R* = 1500), the min-max range and the variance of the scores have increased with each subsequent voting round and revealed expectedly a slightly double-humped data distribution. The final min-max range is eventually 245.86 Elo scores. The plot shows positive correlation with medium to strong relationship between the location and predicted scores which is also confirmed by Pearson correlation coefficient (0.661).

It cannot necessarily be inferred from the entire micro location that the immediate environment of an apartment has similar quality. It is therefore assumed that the two variables could have both a shared and an independent effect on the apartment price, which could also be observed. The Kruskal-Wallis rank sum test indicates a slight difference between the variable micro location and location scores (*p* = 0.05773). In addition, we have incorporated a smoothing function into the scatter plot (Fig. 5), resp. Loess locally weighted regression, which is well suited for determining polynomial effects in smaller data sets. In the present case, the data points indicate an obvious curvature and thus polynomial significance in the relationship between the estimated and predicted scores especially throughout the micro locations with a simple housing value. According to D'Agostino's skewness test applied to location scores, there is no need for its logarithmic transformation with respect to its implementation in the hedonic models.

(b) Additional Test: Evaluation of the Performance of Convnet Trained on Datasets of Different Sizes

With the following experiments we want to evaluate how important the size of the training set is for the training of ConvNet resp. if evaluated network architecture can effectively take benefit of additional training data. Thereby, the amount of training data is directly related to the number of human participants and their repeated trials. We principally expect that more data should be beneficial for the network's performance. Thus, with our experiments we want to quantify the magnitude of the achievable improvement.

We subset *T_r_* into T_r500_ and T_r250_ by random and subsequently train the network on all 3 training sets using same network settings as in the Table 2. To evaluate performance of the three trained networks we use same data set *T_s_* and calculate RMSE as a performance measure. We perform ConvNet training on differently large training sets *T_r_*, _Tr500_ and T_r250_ to see if additional training data improves test performance. The evaluation is performed in on the test set *T_s_* for all training partitions *T_r_*, T_r500_ and *T_r250_*. Quantitative results are summarized in Table 4. To investigate the impact of the dataset size on scores extraction performance we first train EfficentNet-B0 on the largest training set *T_r_* and then repeat the training process with the remaining two smaller training sets. The results show that the performance across the test set decreases when a smaller dataset is used for training. The RMSE decreases by 4.238 and 3.675 prediction errors for larger training sets T_r500_ and *T_r_*, respectively.

(c) Additional Test: Correlation Between the Image Contents and the Location Scores Estimated by Humans

**Table 4:** Performance of the ConvNet model using different amounts of training data.

Training data	Best training epoch	Training loss	Training RMSE	Best val. epoch	Val. loss	Val. RMSE	Test RMSE
788	984	1504.188	38.783	340	1186.14	34.44	39.547
500	993	1657.966	40.718	472	1667.795	40.838	43.222
250	966	1918.37	43.799	394	1875.533	43.307	47.46

With this experiment we would like to infer which specific image areas are decisive for the participants in their subjective assessment of the quality of the immediate residential location. First, we aim at eliciting the proportion of different areas of interest in the image montages. For that purpose, we first apply pixel-level segmentation with pretrained Segnet encoder-decoder network [81] that has been pretrained on the CamVid dataset [82,83] to predict 11 classes of semantic labels. However, we focus in the Table 5 on analysis of 6 classes (Sky, Building, Road, Pavement, Greenery, Fence) as the remaining classes have no contextual relevance for the evaluation of the location quality. Thus, we classify every pixel in an image by predefined classes with the segmentation network, producing a matrix of the same size as input image.

**Table 5:** Correlations and coefficients of determination showing relationship between segmented areas of interest and human estimated location scores.

	Sky	Building	Road	Pavement	Greenery	Fence	Car
Pearson Correlation	0.253	-0.492	-0.086	-0.161	0.534	-0.093	-0.288
Coefficient of Determination	0.064	0.242	0.007	0.026	0.285	0.008	0.083

**Fig. 6:**
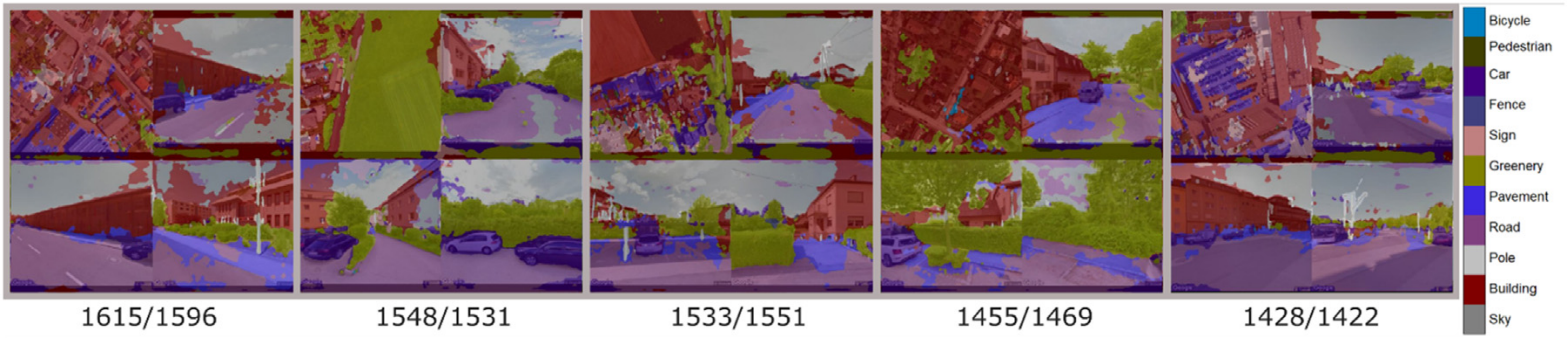
Images of the immediate apartment environment previously shown in Fig. 1 with human estimated and ConvNet predicted location scores as well as segmented areas of interest used for eliciting the focus of choice of participants.

Fig. 6 shows same image montages presented previously in Fig. 1 but with segmented areas of interest and estimated location scores. For this we test each image montage from *Ɗ_i_*, with the Segnet encoder-decoder network [81] and calculate the proportion of pixels of the predicted segmented areas. Subsequently, we investigate the relationship between ratios of the segmented areas and estimated location scores by calculating Pearson correlation coefficient and the coefficient of determination. From the results in the Table 5, it is noticeable that the participants considered sparse building density and foremost the amount of green in the immediate environment as a predominant valorization factor, which is reflected in the correlation of the estimated scores with the segmented green areas in the experimental images (-0.49 resp. 0.53).

## Method discussion

The integration of alternative data extraction methods, as well as the ability to handle alternative data using Machine Learning, can significantly mitigate the difficulties in modeling and provide information from new sources and modalities [56]. Regarding the problem at hand, the benefit of generating human-based estimates and transferring the estimates to a Machine Learning model to produce synthetic data is understandable if an effect of the estimates and predictions on the property price can be confirmed within a potentially applicable model. It is generally accepted that humans, especially those with domain expertise, can perceive complex visual semantics better than ConvNet AI algorithms. The effort for such manual annotations of ground truth justifies, however, the generation of synthetic data in view of the necessary data quantity needed for automated valuation models. Our goal was to show how cohesively the presented methods work on the existing dataset in the presented scenario, without further optimizing them and thereby potentially overfitting them to the data. Hence, we aimed to quantify the degree of generation of additional data that can be achieved, understanding that the effect of additional training data is important in practice to balance the cost of creating additional labeled training data and the expected benefit, i.e., if the expected benefit of additional training data is low, it may not justify the cost of generating annotations and thus additional synthetic data.

## Ethics statements

All procedures performed in studies involving human participants were in accordance with the ethical standards of the University of Applied Sciences Kufstein, Tirol and with the 1964 Helsinki declaration and its later amendments or comparable ethical standards.

## CRediT author statement

All authors contributed to the conception and design of the study. Pre-analyses were conducted by Miroslav Despotovic, Eric Stumpe and Matthias Zeppelzauer. Data collection and processing as well as main analyses were conducted by Miroslav Despotovic. The first draft of the manuscript was written by Miroslav Despotovic, Simon Thaler, Eric Stumpe, David Koch and Matthias Zeppelzauer. Post-analyses were conducted by all authors. All authors have read and approved the final manuscript.

## Declaration of competing interest

The authors declare that they have no known competing financial interests or personal relationships that could have appeared to influence the work reported in this paper.

## Data Availability

The data used in this study is subject to restrictions imposed by the data provider and is therefore not publicly available.
